# Haematological Reference Intervals in a Multiethnic Population

**DOI:** 10.1371/journal.pone.0091968

**Published:** 2014-03-18

**Authors:** Angeli Ambayya, Anselm Ting Su, Nadila Haryani Osman, Nik Rosnita Nik-Samsudin, Khadijah Khalid, Kian Meng Chang, Jameela Sathar, Jay Suriar Rajasuriar, Subramanian Yegappan

**Affiliations:** 1 Department of Haematology, Hospital Ampang, Ampang, Selangor, Malaysia; 2 Department of Community Medicine and Public Health, Universiti Malaysia Sarawak, Kuching, Sarawak, Malaysia; Indian Institute of Toxicology Reserach, India

## Abstract

**Introduction:**

Similar to other populations, full blood count reference (FBC) intervals in Malaysia are generally derived from non-Malaysian subjects. However, numerous studies have shown significant differences between and within populations supporting the need for population specific intervals.

**Methods:**

Two thousand seven hundred twenty five apparently healthy adults comprising all ages, both genders and three principal races were recruited through voluntary participation. FBC was performed on two analysers, Sysmex XE-5000 and Unicel DxH 800, in addition to blood smears and haemoglobin analysis. Serum ferritin, soluble transferrin receptor and C-reactive protein assays were performed in selected subjects. All parameters of qualified subjects were tested for normality followed by determination of reference intervals, measures of central tendency and dispersion along with point estimates for each subgroup.

**Results:**

Complete data was available in 2440 subjects of whom 56% (907 women and 469 men) were included in reference interval calculation. Compared to other populations there were significant differences for haemoglobin, red blood cell count, platelet count and haematocrit in Malaysians. There were differences between men and women, and between younger and older men; unlike in other populations, haemoglobin was similar in younger and older women. However ethnicity and smoking had little impact. 70% of anemia in premenopausal women, 24% in postmenopausal women and 20% of males is attributable to iron deficiency. There was excellent correlation between Sysmex XE-5000 and Unicel DxH 800.

**Conclusion:**

Our data confirms the importance of population specific haematological parameters and supports the need for local guidelines rather than adoption of generalised reference intervals and cut-offs.

## Introduction

The reference interval for Full Blood Count (FBC), one of the commonest baseline investigations, is usually from manufacturers of haematology analysers or publications. However, as FBC parameters are known to vary between populations, ethnicities, age and gender, a population specific interval is clinically sound and desirable [1]–[10] (Table 1).

**Table 1:** Comparison of Reference Intervals of various populations.

Parameter	Gender	Gaza Strip	Germany	US	UK	South India	Ghana
		Mean (Range)	Range	Mean	Mean (Range)	Mean (Range)	Range
Haemoglobin (g/dL)	Male	14.79 (12.67–16.91)^a^	12.57–16.44^c^	W 15.17^d^	15 (13–17)^h^	14.43 (11.13–17.40)^i^	11.3-16.4^j^
		14.52 (12.08–16.96)^b^		B 14.25^d^			
				W 14.94^e^			
				B 14.19^e^			
	Female	12.39 (9.99–14.79)^a^	11.28–14.66^c^	W 13.76^d^	13.5 (12–15)^h^	12.14 (9.22–14.67)^i^	8.8–14.4^j^
		12.48 (9.92–15.04)^b^		B 13.07^d^			
				W 13.66^e^			
				B 12.89^e^			
RBC (10^12^/L)	Male	5.1 (4.24–5.96)^a^	3.94–5.43^c^	4.18–5.86^f^	4.7 (3.8–5.5)^h^	5.10 (4.01–6.04)^i^	3.79–5.96^j^
		4.99 (3.99–5.99)^b^		3.57–5.67^g^			
	Female	4.48 (3.66–5.30)^a^	3.64–4.93^c^	3.64–5.2^f^	4.2 (3.6–4.8)^h^	4.39 (3.44–5.30)^i^	3.09–5.30^j^
		4.71 (3.87–5.55)^b^		3.51–5.34^g^			
Hematocrit (%)	Male	44.43 (38.15–50.71)^a^	37–48^c^	38.7–51.4^f^	45 (40–50)^h^	41.63 (33.07–49.97)^i^	33.2–50.5^j^
		43.81 (36.55–51.07)^b^		33.9–50.9^g^			
	Female	37.84 (30.96–44.72)^a^	33–43^c^	32.0–45.9^f^	40 (35–45)^h^	35.85 (29.00–42.78)^i^	26.4–45.0^j^
		38.84 (28.58–49.10)^b^		32.8–47.0^g^			
Platelets (10^9^/L)	Male	270.28 (143.22–397.34)^a^		152–386^f^	275 (120–410)^h^	242.26 (148.32–404)^i^	88–352^j^
		273.14 (124.96–421.32)^b^		124–384^g^			
	Female	303.73(153.29–454.17)^a^	129–327^c^	168–441^f^		261.6 (146.90–408.78)^i^	89–403^j^
		318.13 (144.41–491.85)^b^		155–428^g^			

Gaza Strip (N = 50,127)[1].

Germany(N = 2967)[6].

US (N = 7664) [4], (N = not available)[5], (N = not available)[8].

UK (N = 700)[2].

South India(N = 500)[3].

Ghana (N = 691)[7].

a 19–45 years old.

b >45 years old.

c 20–81 years old.

d 50–59 years old.

e 60–69 years old.

f 19–65 years old.

g 66 years and above.

h 16–91 years.

i 18–70 years.

j 18–59 years.

W = White B = Black.

FBC reference intervals in use in Malaysia are generally from non-Malaysian populations; the only locally derived interval was based on 199 subjects, 45 years or younger and of unspecified race [11]. As a reference interval for adults should be derived from an adequate number of subjects, all ages and all ethnicities (if in a multiracial population), we embarked on this study to establish a comprehensive reference interval for Malaysian adults. Our hypothesis was that there would be race, gender and age associated differences.

## Materials and Methods

### Subject recruitment

Based on the standard deviation values of haemoglobin reported by Roshan et al [11] and setting the type I error and type II error at 0.05 and 0.20 respectively, we calculated a minimal sample size of 1200 subjects (considering 0.10 g/dL as the minimal significant difference between the sample mean and the true population mean, and an attrition rate of 20%). To facilitate the subject recruitment process, we divided the population into 12 subgroups, based on gender (males, females), ethnicity (Malay, Chinese, Indian) and age group (above and below 60 years for males; pre-menopausal and post-menopausal for females). In each subgroup, we tried to recruit at least 120 subjects but the number could only be achieved in female subjects and Chinese male subjects less than 60 years old. The reasons for not achieving the target of 120 subjects in the other subgroups were exclusion due to the presence of exclusion criteria and lack of voluntary participation by the population.

Subjects were recruited through voluntary participation in a series of screening programs conducted at premises of various companies and government departments and at community gatherings in and around the Klang Valley, Selangor and the contiguous states of Negeri Sembilan, Perak and Pahang in Malaysia from September 2011 to December 2012.

Inclusion criteria for the study were all male and non-pregnant female Malaysian citizens 19 years or older consenting to participate in the study. Subjects who had fever in the past week, history of any malignancy, immune thrombocytopenia, thalassemia, jaundice, renal disease, any transfusion or blood donation in the preceding six months were excluded from the study.

### Data collection

Basic demographic data and information on current medical history, medication intake, blood donation, blood transfusion, smoking status, menopause status and vegetarian status were collected using a self-administered questionnaire.

Two tubes of 3 ml of blood in K2 EDTA and a 5 ml plain tube were drawn from all qualified subjects following written informed consent. Serum was separated and stored at –80C for serum ferritin, soluble transferrin receptor and C-reactive protein (CRP) assays.

### Laboratory testing

FBC was performed on the Sysmex XE 5000 (Sysmex, Kobe, Japan) and Unicel DxH 800 (Beckman Coulter, USA) analysers within 6 hours of sampling following International Council for Standardization in Haematology (ICSH) guidelines [12], [13]. Smears were performed in all subjects on the SP1000i automated slide maker (Sysmex, Kobe, Japan) and reviewed by a haematopathologist. H inclusions were detected by supravital brilliant cresyl blue (BCB) staining (Merck Millipore, Darmstadt, Germany). Haemoglobin analysis was performed on the Capillarys 2 (Sebia, France).

Serum ferritin (Modular E170, Roche, Switzerland) and soluble transferrin receptor (Cobas Integra 400, Roche, Switzerland) assays were performed on all subjects with haemoglobin >10 g/dL. C-reactive protein (AU 480, Beckman Coulter, USA) was assayed in subjects with increased serum ferritin [males >400 ng/mL, females >150 ng/mL].

Analysers were calibrated and maintained according to the manufacturer's instructions. Internal quality control (QC) was performed and samples were assayed only if all QC criteria were fulfilled. Our laboratory is enrolled in the Royal College of Pathologists of Australasia (RCPA) proficiency programs for full blood count, reticulocyte count, manual differential, automated differential, haemoglobin analysis and serum ferritin. Assay precision was monitored by internal QC assessment and accuracy was determined based on external quality control performance.

### Data analysis

Subjects were excluded from the reference interval calculation if they fulfilled any of the following criteria: haemoglobin <10 g/dL, presence of South East Asian Hereditary Ovalocytosis (SEAHO) or H inclusions, smokers (current and past), serum ferritin ≤13 ng/mL, HbA_2_ ≥3.5, any Hb variant, mean corpuscular haemoglobin (MCH) <27 pg (on both analysers), subjects with increased serum ferritin (males ≥400 ng/mL, females with ≥150 ng/mL and C-reactive protein ≥10 mg/dL) and incomplete demographic data. FBC parameters of male smokers were compared with male non-smokers; however a similar comparison was not performed in females as there were too few female smokers.

All hematological parameters in this study were tested for normality using the Shapiro Wilk test in total and according to the predefined subgroups. The measures of central tendency (mean, median, mode) and dispersion (standard deviation (SD), range, inter-quartile range), and other point estimates (values at 2.5th, 25.0th, 75.0th and 97.5th percentiles) were calculated for all quantitative hematological values in total and according to subgroups. Differences between each hematological value found in this study and the established values were tested using analysis of variance method. For non-normally distributed parameters, statistical analysis was carried out using the corresponding non-parametric method. The reference interval was defined as mean ± 1.96SD for normally distributed data or values at 2.5 percentile and 97.5 percentile for non-normally distributed data. Data cleaning was performed using Statistical Package for Social Sciences (SPSS) version 19 software and statistical analysis was performed using STATA Intercooled version 11 software.

### Ethical consideration

This study was approved by the Medical Research Ethics Committee of the Ministry of Health, Malaysia and registered with the National Medical Research Register (Research ID 10-277-5480). Written informed consent was obtained from all participants before data collection.

## Results

Of the 2725 subjects who were recruited complete data was available in 2440. One thousand three hundred seventy six (56.4%) fulfilled all inclusion criteria and were selected for reference interval calculation. Malays, Chinese and Indians each constituted about a third of the 1376 subjects, two-thirds were female and the mean age of menopause was 49 years (Table 2).

**Table 2:** Demography of normal subjects included in reference interval calculation.

	Malay, n (%)	Chinese, n (%)	Indian, n (%)	Total	Age (years), (mean SD); median
**All Males**	123 (26.2)	191 (40.7)	155 (33.0)	469	48.5(18.2); 51
Men <60	83 (26.7)	122 (39.2)	106 (34.1)	311	38.2 (12.9); 35
Men ≥60	40 (25.3)	69 (43.7)	49 (31.0)	158	68.9 (5.5); 68
**All Females**	307 (33.8)	325 (35.8)	275 (30.3)	907	45.9 (17.7); 48
Premenopausal	179 (37.0)	186 (38.4)	119 (24.6)	484	32.0 (9.0); 30
Postmenopausal	128(30.3)	139 (32.9)	156 (36.9)	423	62.0 (9.0); 61
**Total**	430 (31.3)	516 (37.5)	430 (31.3)		

Forty four percent (1064/2440) of the recruited group were excluded. Forty three percent of males and one percent of females were current or past smokers; about one percent had HbA2 ≥3.5 and 2.7% showed Hb variants and SEAHO was observed in 6.7% of Malays. Other exclusion factors were haemoglobin <10 g/dL, CRP ≥10 mg/dL and reactive leukocytosis. Some of these were more frequent in older males resulting in fewer qualified subjects in this group. The prevalence of anaemia, iron deficiency and iron deficiency anaemia (among subjects in whom serum ferritin was tested) based on the haemoglobin lower limits of this study is shown in Table 3.

**Table 3:** Incidence of anaemia, iron deficiency and iron deficiency anaemia.

	Anaemia, n (%)	Iron deficiency,n (%)	Iron deficiency anaemia, n (%)
Males <60, n = 642	35(5.5)	8(1.25)	7(1.1)
Males >60, n = 351	25(7.1)	5(1.4)	5(1.4)
Premenopausal women, n = 784	99 (12.6)	154 (19.6)	68 (8.7)
Postmenopausal women, n = 594	73(12.3)	18(3)	18(3)

There was excellent correlation between the two analysers for all parameters except mean corpuscular haemoglobin concentration (MCHC) and platelet distribution width (PDW) for which correlation was good and basophils for which correlation was poor (data not shown). Hence, the reference range was calculated based on our primary analyzer, the XE 5000 (Sysmex, Kobe, Japan) except for those parameters unique to the DxH 800 (Beckman Coulter, USA).

As we did not find any clinically significant difference between Malays, Chinese and Indians for any parameter, data was combined. Age and gender specific intervals were obtained only for haemoglobin, red blood cell (RBC) count, hematocrit, platelet count and serum ferritin; none of the other parameters showed any clinically significant age or gender associated variation. These intervals are shown in Table 4, 5.

**Table 4:** Reference intervals from this study compared with previous study.

Parameter	Mean	Median	Reference interval	Roshan *et al*. [11]
			(mean±1.96SD)	mean, (mean±2SD)
Haemoglobin (g/dL)				
Males <60	15.45	15.5	13.5–17.4	14.27, 12.01–16.53
Males >60	14.36	14.3	11.8–16.9	
Females	13.33	13.3	11.6–15.1	11.83, 9.81–13.85
RBC Count (10^12^/L)				
Males <60	5.24	5.23	4.53–5.95	5.12, 4.18–6.06
Males >60	4.74	4.75	3.86–5.62	
Females	4.54	4.55	3.87–5.21	4.34, 3.52–5.16
Haematocrit (%)				
Males <60	45.3	45.5	40.1–50.6	43.62, 37.48–49.76
Males >60	42.3	42.2	35.7–48.9	
Females	40	40	35.1–44.9	37.08, 31.76–42.4
MCV (fL)	88	87.9	80.6–95.5	M 87.32, 78.90–95.74
				F 85.99,77.49–94.49
MCH (pg)	29.6	29.6	26.9–32.3	M 28.24, 25.38–31.10
				F 27.99,24.75–31.23
MCHC (g/dl)	33.6	33.6	31.9–35.3	M 32.7, 30.58–34.82
				F 31.9,29.43–34.35
RDW-SD (fl)	42.8	42.7	37.5–48.1	M 41.14, 35.52–46.76
				F 41.63,35.71–47.55
RDW-CV (%)	13.4	13.3	12–14.8	M 13.18, 11.20–15.16
				F 13.23,11.37–15.09
Total WBC (10^9^/L)	7.724	7.500	4.078–11.370	M 6.74,3.78–9.7
				F 6.73,3.37–10.09
Neutrophils (10^9^/L)	5.538	5.525	3.929–7.147	M 3.76,1.58–5.94
				F 3.81,1.55–6.07
Lymphocytes (10^9^/L)	3.327	3.325	1.847–4.807	M 2.18,1.14–3.22
				F 2.17,1.05–3.29
Monocytes (10^9^/L)	0.763	0.740	0.385–1.141	M 0.41,0.15–0.67
				F 0.42,0.1–0.74
Eosinophils (10^9^/L)	0.329	0.270	0–0.827	M 0.18,0.08–0.28§
				F 0.15,0.03–0.27§
Basophils (10^9^/L)	0.044	0.040	0–0.095	M 0.03,0.01–0.05§
				F 0.03,01–0.05§
Platelets 10^9^/L				
Males	246	240	142–350	254.9, 166–376
Females	285	283	171–399	275.2, 157–410
Ferritin* (ng/mL)				
Males	-	143.5**	30.7–579.2**	114.59(55.7–173.48)§
Premenopausal	-	51**	15–234.5**	51.10(14.45–87.75) §
Postmenopausal	-	120**	22–475.2**	

M =  male, F =  female.

* Median, range 2.5-97.5 centiles.

** Ferritin ng/mL<13 excluded.

§ Reference interval  =  mean±SD.

**Table 5:** Research Parameters, sTfR and HbA2 intervals for this study.

Parameter	Mean	Median	Reference interval (mean±1.96SD)
RetHe (pg)	34.8	34.8	30.7–38.9
Retic (%)	1	1	0.4–1.6
RPI	0.82	0.8	0.1–1.5
IRF (%)	4.2	3.7	0–8.9
%HypoHe	0.46	0.3	0–1.3
%MicroR	1.8	1.5	0–3.8
%HyperHe	1.3	1.3	0.9–1.7
%MacroR	7	6.8	4–10
LHD%	5	3.9	0–13.8
RSf (fL)	98.9	98.5	89.7–108.1
MAF	12.2	12.1	9.8–14.7
MRV (fL)	109	108.95	93.9–124.1
IPF%	1.8	1.5	0–4
MPV (fL)	10.4	10.3	8.9–11.9
sTfR (mg/L)	3.2	3.1	1.4–5
HbA2	2.6	2.6	2.13–3.07

HbA2 ≥3.5 excluded.

## Discussion

We have ascertained FBC parameters in 1376 healthy Malaysian adults (Table 4, 5) using the XE 5000 (Sysmex, Kobe, Japan) and DxH 800 (Beckman Coulter, USA) analysers. This is the largest number of subjects assessed for FBC reference interval in Malaysia, the first multiethnic study and the only one to compare two analysers.

We found that there were clinically relevant differences compared to other populations, emphasising the necessity for population specific reference intervals [1]–[10] (Table 1). Females and younger males in Chennai (South India) and the Gaza Strip have lower haemoglobin than adults in Malaysia while older males have slightly higher levels. In Ghanaian male and female adults, haemoglobin levels and the 2.5^th^ percentile for RBCs and platelets are significantly lower than in our subjects. The differences between populations in the US, UK and Germany and our subjects do not appear to be clinically significant. Though ethnicity cannot be ruled out as a contributor to these differences, it is less likely to be significant. It is probable that other factors like the environment and nutritional status may play a greater role. This is supported by the differences between South Indians in Chennai and Malaysian Indians even though both populations are from the same ethnic group.

However, our findings also differ significantly from the only locally published reference values [11]. This is most likely due to a greater number of subjects (1376 vs 199), exclusion of confounding factors like thalassemia trait and iron deficiency, and inclusion of older subjects in this study affirming that reference intervals studies should be broad based in recruitment but with appropriate exclusion criteria.

We confirm that there are clinically relevant gender and age but not race related differences for haemoglobin, RBC count, hematocrit and platelet count; however, none of the other parameters showed significant variation by age, gender or ethnicity.

Like others, we found that haemoglobin, RBC count and hematocrit decrease with age in males; but in contrast to other populations, we did not find a similar trend comparing premenopausal and postmenopausal women. In line with our findings, a study of Nigerian women reported no statistically significant difference for haemoglobin between premenopausal and postmenopausal women [15].

Malaysian blood donation guidelines recommend a haemoglobin ≥12.5 g/dL in prospective donors; but given this study's lower limit for haemoglobin (11.6 g/dL in women, 11.8 g/dL in men >60, 13.5 g/dL in men <60), these guidelines should be reviewed in favour of a gender and age appropriate cutoff [16]. We suggest that women with haemoglobin ≥11.6 g/dl be considered as potential blood donors while younger men with haemoglobin <13.5 g/dL be disqualified. Otherwise not only would there be exclusion of *qualified* women but also inclusion of *anaemic* younger men. Likewise, the WHO cutoff for mild anaemia (haemoglobin 11–11.9 g/dL in non-pregnant female and 11–12.9 g/dL in male) would erroneously designate 3.6% of females and 13.3% of older males as anaemic, while missing 2.3% of younger males who are anaemic [17], [18]. Hence we recommend instead the adoption of haemoglobin cutoffs from this study for the diagnosis of mild anaemia at least in urban Malaysian adults.

This study includes the largest number of subjects assessed for the advanced RBC parameters like IRF (Immature Reticulocyte Fraction), %Hypo-He, MicroR, %Hyper-He, MacroR, Reticulocyte Hemoglobin (Ret-He), Mean Reticulocyte Volume (MRV), Red blood cell size factor (RSf), Low Hemoglobin Density (LHD%), Microcytic Anaemia Factor (MAF), soluble transferrin receptor, serum ferritin and HbA_2_ (Table 4,5). Traditionally anaemias have been classified based on mean corpuscular volume (MCV); however, the distinction amongst microcytic anaemias is not clear cut especially in populations with a significant prevalence of thalassemia carriers. Unlike traditional parameters like MCV or MCH which reflect all red blood cells, %Hypo-He, MicroR, %Hyper-He and MacroR assess subpopulations [19], [20]. %Hypo-He and %Hyper-He are the percentage of RBCs with haemoglobin content <17 pg and >49 pg respectively, while MicroR and MacroR are the percentage of cells with MCV of <60 fL and >120 fL respectively. Ret-He and %Hypo-He have been suggested to be reliable indicators of functional iron deficiency as they reflect iron availability compared to ferritin which is a measure of iron stores not supply [20], [21], [22]. These advanced RBC parameters could serve as inexpensive surrogate measures of iron restricted erythropoiesis and iron availability especially in the background of inflammation and erythropoietin therapy. Wider usage of these parameters could also lead to better classification, monitoring and management of anaemia.

The number and distribution of leukocytes in our subjects differs from other populations. However, in agreement with the literature, males have fewer neutrophils than females (count increasing with age in males and reducing in females), while females have a higher lymphocyte count (in contrast to neutrophils, lymphocyte count increases with age in females but decreases in males). In postmenopausal women, the 97.5th percentile for lymphocyte count is >5000×10^6^/μl suggesting that the current designation of lymphocytosis as >5000×10^6^/μl may not be a valid criterion in all subjects (data not shown). Males have greater numbers of monocytes and eosinophils. But as the 97.5th percentile for all of our subjects is >800×10^6^/μl monocytes and >500×10^6^/μl eosinophils, the designation of monocytosis and eosinophilia in Malaysians similarly requires review.

Similar to other studies, we found that females have higher platelet counts than males, and that count decreases with age in both [14]. However, defining thrombocytopenia as <150,000×10^6^/μl may not be optimal in older males as the –2SD in this group is 128,000×10^6^/μl (data not shown). The interval for IPF (Immature Platelet Fraction) is consistent with the literature [23], [24], [25]. IPF, an indicator of thrombopoiesis, can be used to evaluate etiology of thrombocytopenia, monitor hematopoietic regeneration post-transplant and chemotherapy and in deciding the timing and necessity of platelet transfusion, with the potential for, and objective of, reducing transfusions.

The interval for HbA_2_ differs from a recent locally published range (2.25–3.75 versus 2.13–3.07 this study) [26]; however they sampled only 154 normal subjects and we excluded all subjects with HbA_2_ ≥3.5. The interval for soluble transferrin receptor is similar to that provided by the kit manufacturer except that we do not find a significant difference between the genders.

Applying the derived haemoglobin interval in a post hoc manner to the entire sample cohort, we found that in apparently healthy Malaysian adults, the prevalence of anaemia in women was twice that of men and iron deficiency anaemia (IDA) was thrice as frequent in premenopausal compared to postmenopausal women (Table 4). About 70% of all anaemia in premenopausal females, 24.2% in postmenopausal females and 20% in males is attributable to Iron deficiency. These figures compare with local prevalence [27]; however, prevalence rates for iron deficiency and iron deficiency anaemia in this study may not be an accurate reflection of the population as ferritin was performed in a majority of subjects (87.8%) but not all.

In contrast to other studied populations we did not find clinically significant differences in haemoglobin, RBC count and total WBC count between smokers and non-smokers. We agree with Milman *et al* in not recommending a separate reference interval for smokers [28].

Our reference interval may not be representative of all Malaysians as ours was convenience sampling and subjects were predominantly urban; however, at least 25% of the total Malaysian population resides in our catchment area and more than 70% of Malaysians are urban [29]. Also, the origin of our subjects based on place of birth includes all regions of Malaysia.

In summary, we have obtained a reference interval in apparently healthy Malaysian adults for all FBC parameters currently available in two high end analysers. These findings support a review of the local use of the WHO haemoglobin cutoff for diagnosis of anemia and the cutoff for the selection of blood donors, and the threshold used for the designation of lymphocytosis, monocytosis, eosinophilia and thrombocytopenia in Malaysian adults. We suggest that in Malaysia, blood donors are not an appropriate group to use for FBC reference intervals. Finally, a race based reference interval for FBC in Malaysian adults is not supported by our data.
